# A combination of *Lactobacillus mali* APS1 and dieting improved the efficacy of obesity treatment via manipulating gut microbiome in mice

**DOI:** 10.1038/s41598-018-23844-y

**Published:** 2018-04-18

**Authors:** Yung-Tsung Chen, Ning-Sun Yang, Yu-Chun Lin, Shang-Tse Ho, Kuan-Yi Li, Jin-Seng Lin, Je-Ruei Liu, Ming-Ju Chen

**Affiliations:** 1 Institute of Biotechnology, National Taiwan University, Taipei City, 106 Taiwan; 2 Agricultural Biotechnology Research Center, Academia Sinica, Taipei City, 115 Taiwan; 3 Department of Animal Science and Technology, National Taiwan University, Taipei City, 106 Taiwan; 4 Taiwan Livestock Research Institute, Council of Agriculture, Executive Yuan, Tainan City, 71246 Taiwan; 5 SynbioTech Incorporation, Kaohsiung City, 821 Taiwan

## Abstract

The difficulty of long-term management has produced a high rate of failure for obesity patients. Therefore, improving the efficacy of current obesity treatment is a significant goal. We hypothesized that combining a probiotic *Lactobacillus mali* APS1 intervention with dieting could improve the efficacy of obesity and hepatic steatosis treatment compared to dieting alone. Mice were fed a high-fat diet for 6 weeks and then treated with: saline + normal diet and APS1 + normal diet (NDAPS1) for 3 weeks. NDAPS1 accelerated body weight loss and reduced caloric intake and fat accumulation. The fecal microbiome showed that accelerating weight loss by NDAPS1 resulted in restoring intestinal microbiota toward a pre-obese state, with alteration of specific changes in the obesity-associated bacteria. APS1 manipulated the gut microbiome’s obesity-associated metabolites, followed by regulation of lipid metabolism, enhancement of energy expenditure and inhibition of appetite. The specific hepatic metabolites induced by the APS1-manipulated gut microbiome also contributed to the amelioration of hepatic steatosis. Our results highlighted a possible microbiome and metabolome that contributed to accelerating weight loss following treatment with a combination of APS1 and dieting and suggested that probiotics could serve as a potential therapy for modulating physiological function and downstream of the microbiota.

## Introduction

An alarming increase in the prevalence of overweight and obesity worldwide has occurred in the past decade^1^. Obesity usually accompanies a high risk of metabolic disorder and several complications, such as multiple disabilities, diabetes, heart disease and cancers. Physical exercise and dieting have been considered the first-line therapeutic treatment of obesity and obesity-associated metabolic disorders^2^. Recent studies have focused on dietary modifications, such as calorie restriction^3^ or antioxidant intervention^4^, to limit and protect against high fat diet (HFD)-induced weight gain and lipogenesis *in vivo*. Nevertheless, on a spectrum of medical and scientific supports, long-term management intending to attenuate or reverse the prevalence of obesity still fails to fulfill expectations. Therefore, it is urgent to investigate effective treatment that could enhance the effects of current management protocols for obesity.

The gut microbiome plays a critical role affecting metabolic homeostasis and risk of developing obesity with its metabolic complications^5,6^. The compositional and functional microbiome alterations, such as proportions of the two predominant bacteria, Firmicutes and Bacteroidetes^7^, and intestinal endotoxin-producing bacteria^8^, have been suggested to contribute to the pathogenesis of obesity in both animal models and humans. Fecal microbiota transplantation from obese mice into germ-free mice led to an increase in body weight gain and energy absorption^9^, also indicating that dysbiosis of the gut microbiome is associated with pathological activities in obesity. Moreover, accumulating evidence has demonstrated that changes in the gut microbiome are linked to a variety of factors related to host metabolism, such as the obesity-related metabolome^10^, regulation of appetitive gut hormones^11^, fermentation of intestinal short chain fatty acids (SCFAs)^12^, and progress of nonalcoholic fatty liver disease^5^ in obesity.

Dietary changes have been demonstrated to be central drivers of microbiome composition and function. Probiotics are generally recognized as safe for human use^13^ and have been demonstrated to have beneficial effects on improvement of obesity through manipulating gut microbiota composition^14^. Specific probiotic strain interventions also showed protective abilities against hepatic steatosis by inhibiting fatty acid synthesis and suppressing inflammation *in vivo*^15,16^. However, most probiotic studies were focused on the protective effect on obesity. There have been few studies reporting the efficacy of probiotics in combination with dietary modifications on the improvement of obesity. The mechanisms underlying probiotic-induced weight-loss with dieting remain to be determined.

In our previous study, we demonstrated that administration of *Lactobacillus mali* APS1 (APS1), which was isolated from sugary kefir grain, reduced body weight gain and inflammatory activity and changed body composition in HFD-induced obese mice^17^. Thus, based on our previsions findings, we hypothesized that combining APS1 intervention and dieting could accelerate body weight loss and improve obesity-related pathologies comparing with dieting along. To test the hypothesis, we evaluated the impact of a combination of APS1 and dieting on body weight control and obesity-related metabolome and structure of the gut microbiota *in vivo* and gained an understanding of the mechanisms of obesity treatment. The mechanisms underlying the probiotic-induced weight-loss with dieting were also evaluated through analysis of the microbiome and metabolome. Our findings indicated that the combination of probiotic APS1 and dieting might be a potential alternative to enhance the efficacy of obesity management.

## Results

### APS1 accelerated body weight loss, reduced caloric intake, and lowered fat mass in obese mice on a diet

**Figure 1:**
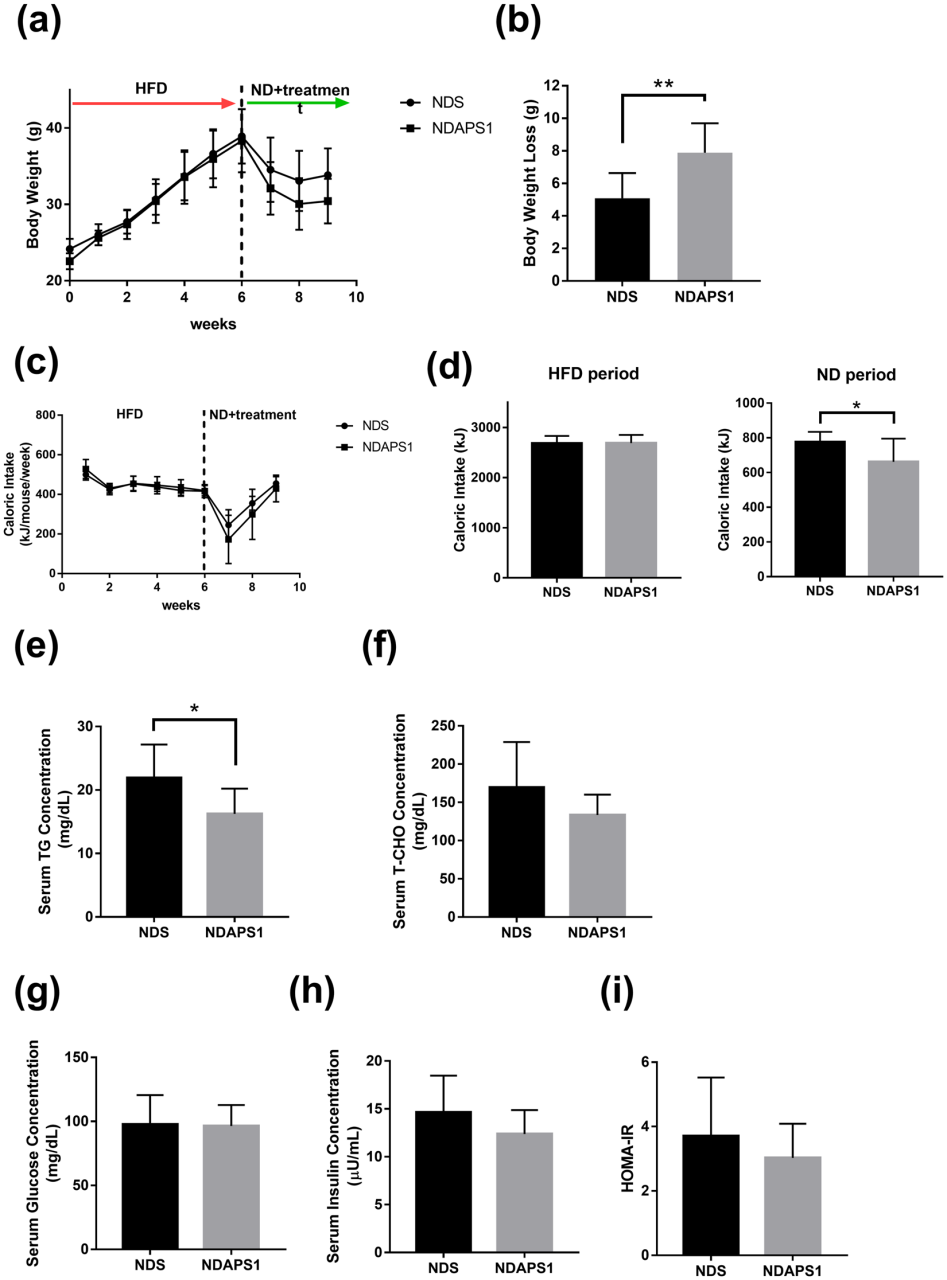
NDAPS1 accelerated body weight loss compared to NDS in preexisting obese mice after dieting. Effect of NDAPS1 on (**a**) body weight, (**b**) body weight loss, (**c**,**d**) caloric intake, (**e**) serum triglyceride, (**f**) total cholesterol, (**g**) glucose, (**h**) insulin levels and (**i**) HOMA-IR in preexisting obese mice are shown. n = 8 per group. The data are expressed as the mean ± SD. Statistical analysis was conducted by using unpaired two-tailed student’s t-test. *P < 0.05, **P < 0.01, ***P < 0.001.

**Figure 2:**
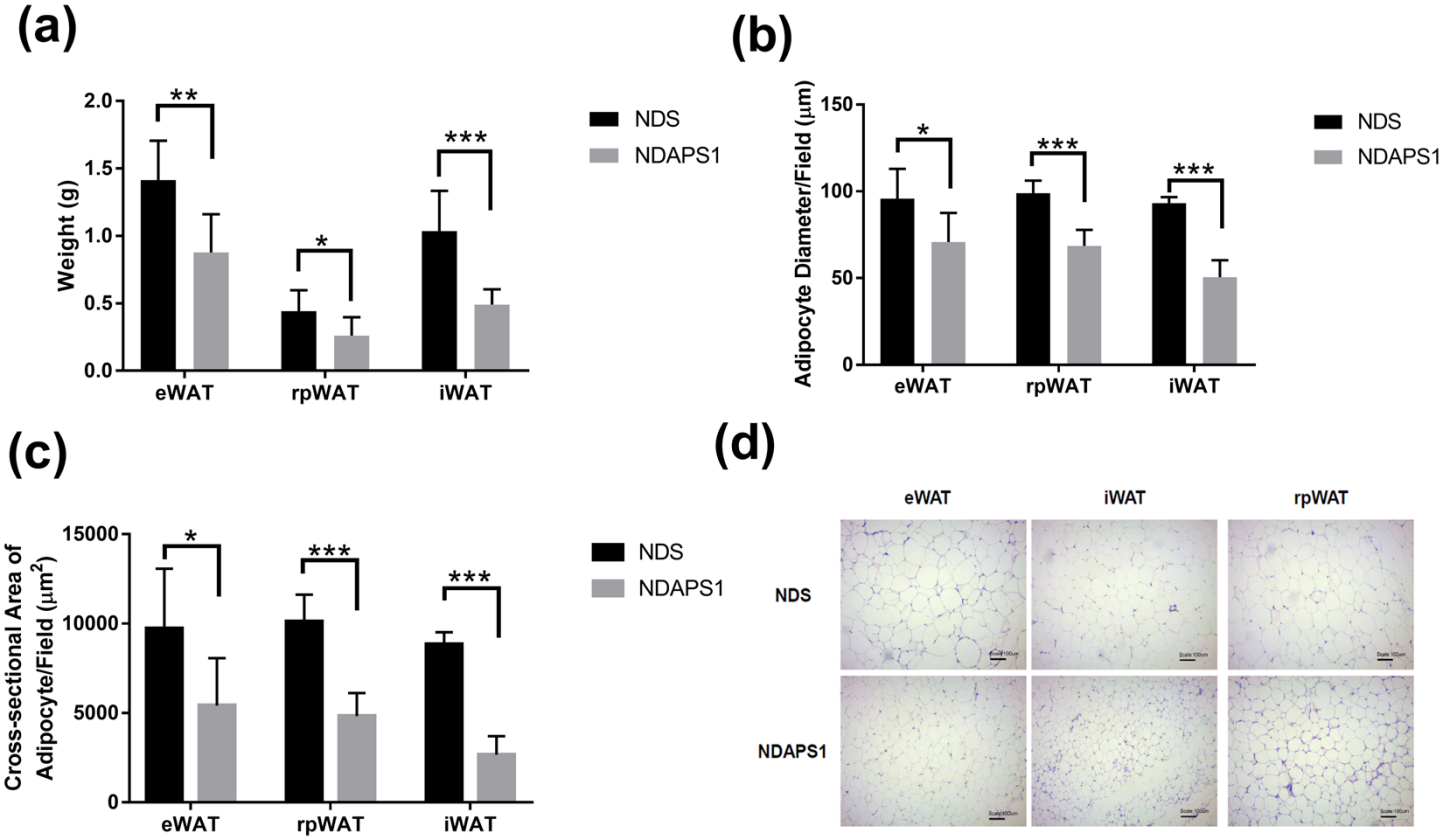
NDAPS1 reduced fat accumulation in preexisting obese mice after dieting. NDAPS1 reduced (**a**) WAT weight, (**b**) diameter and (**c**) cross-sectional area of adipocytes. (**d**) Representative histological sections of WAT from mice treated with NDS and NDAPS1 were stained with H&E staining. Scale bar indicates 100 μm. n = 8 per group. The data are expressed as the mean ± SD. Statistical analysis was conducted by using unpaired two-tailed student’s t-test. *P < 0.05, **P < 0.01, ***P < 0.001.

To examine the effect of APS1 intervention on physiological features of preexisting obesity after dieting, test mice were exposed to an HFD first and then subsequently fed an ND. Our results indicated that body weight gain and metabolic disorder syndromes developed during primary exposure to an HFD followed by weight reduction during exposure to an ND in all test mice (Fig. 1a). APS1 intervention (NDAPS1 group) produced significantly higher weight loss (7.89 ± 1.69 g) when compared with saline treatment (NDS) (5.09 ± 1.45 g) after exposure to an ND (*P* < 0.01) (Fig. 1b). Acceleration of weight loss during the dieting stage in the NDAPS1 group was associated with a decrease in cumulative caloric intake. The cumulative caloric intake of the NDAPS1 group was significantly reduced compared to that of the NDS group (*P* < 0.05) (Fig. 1c,d). Moreover, compared to the NDS group, the NDAPS1 group showed decreases in the serum TG concentration (by 25.83%, *P* < 0.05) (Fig. 1e), weights of eWAT (by 37.93%, *P* < 0.01), rpWAT (by 41.24%, *P* < 0.05) and iWAT (by 52.53%, *P* < 0.001) (Fig. 2a), adipocyte diameters of eWAT (by 26.07%, *P* < 0.05), rpWAT (by 30.70%, *P* < 0.001) and iWAT (by 45.71%, *P* < 0.001) (Fig. 2b), and cross-sectional areas of adipocytes in eWAT (by 44.24%, *P* < 0.05), rpWAT (by 52.21%, *P* < 0.001) and iWAT (by 69.77%, *P* < 0.001) (Fig. 2c,d), but not the serum total cholesterol concentration (*P* > 0.05) (Fig. 1f). The level of fasting glucose showed no difference between the NDS and NDAPS1 groups (*P* > 0.05) (Fig. 1g). A tendency to decrease the levels of fasting insulin and HOMA-IR in the NDAPS1 group was observed in this study but there was no significant difference between two groups (*P* > 0.05) (Fig. 1h,i). Overall, these results indicated that APS1 intervention accelerated body weight loss and reduced fat mass in preexisting obese mice on a diet.

### APS1 ameliorated hepatic steatosis in preexisting obese mice after dieting

**Figure 3:**
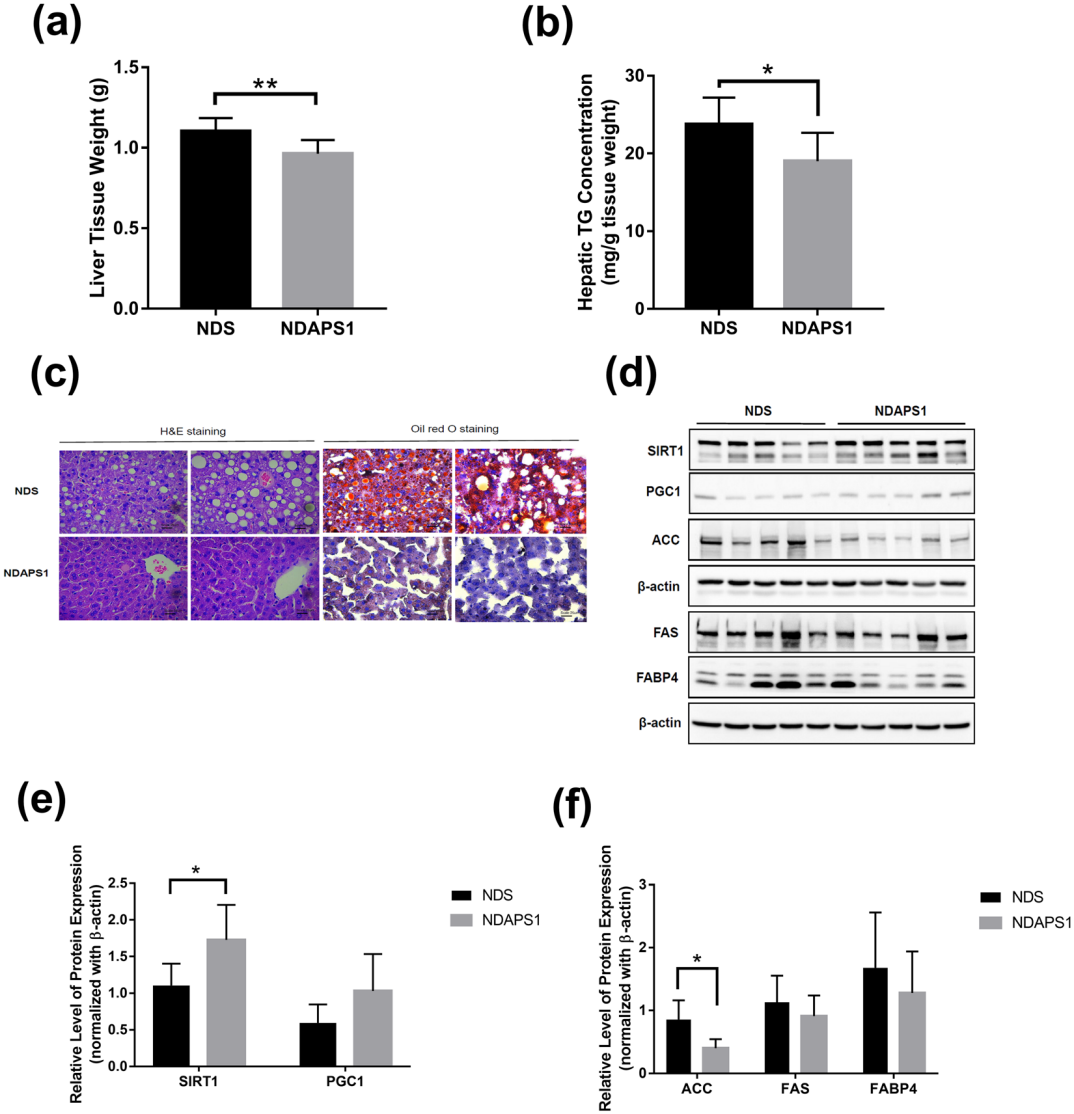
NDAPS1 reduced hepatic steatosis in preexisting obese mice after dieting. (**a**) NDAPS1 reduced liver weight. (**b**) NDAPS1 reduced hepatic TG levels. (**c**) Representative histological sections of liver tissues from mice treated with NDS and NDAPS1 were stained with H&E and Oil Red O staining. Scale bar indicates 25 μm. (**d**) Representative Western blot analysis for expression of SIRT1, PGC1, ACC, FAS, FABP4 and β-actin proteins in liver tissues of NDS and NDAPS1-treated mice. The cropped blots are displayed in the figure, the cropping line is delineated with the black frame line. Un-cropped full length blots are presented in Supplementary Figure S4. (**e**,**f**) Quantitative Western blot analysis by ImageJ software. n = 8 per group. The data are expressed as the mean ± SD. Statistical analysis was conducted by using unpaired two-tailed student’s t-test. *P < 0.05, **P < 0.01.

We further evaluated the effect of APS1 intervention on the improvement of hepatic steatosis in preexisting obese mice after dieting. The NDAPS1 group had significantly lower liver tissue weight (*P* < 0.01) (Fig. 3a) and liver TG level (*P* < 0.05) (Fig. 3b) compared with the NDS group. Reducing the incidence of hepatocyte vacuolation and suppressing accumulation of lipid droplets in liver tissue was also observed in the NDAPS1 group by histological H&E staining and Oil Red O staining (Fig. 3c).

The molecular mechanism involving APS1-ameliorated hepatic steatosis including sirtuin (SIRT) 1, peroxisome proliferator-activated receptor gamma coactivator 1 (PGC1), lipid synthesis factors (ACC, FAS) and fatty acid transporter FABP4 was investigated by immunoblot assay (Fig. 3d–f). The results indicated that the NDAPS1 group showed significantly higher expression of SIRT1 (*P* < 0.05) and lower ACC (*P* < 0.05) in the liver tissue of mice compared with the NDS group. The mice that received the APS1 intervention also showed a trend of increasing expression of PGC1 and decreasing expression of FAS and FABP4 in liver tissue. These results suggested that the preexisting obese mice in the APS1 intervention not only showed accelerated weight loss after dieting but also showed enhanced amelioration of hepatic steatosis through regulation of expression of lipogenesis molecules.

### APS1 manipulated obesity-associated gut microbiota in preexisting obese mice after dieting

**Figure 4:**
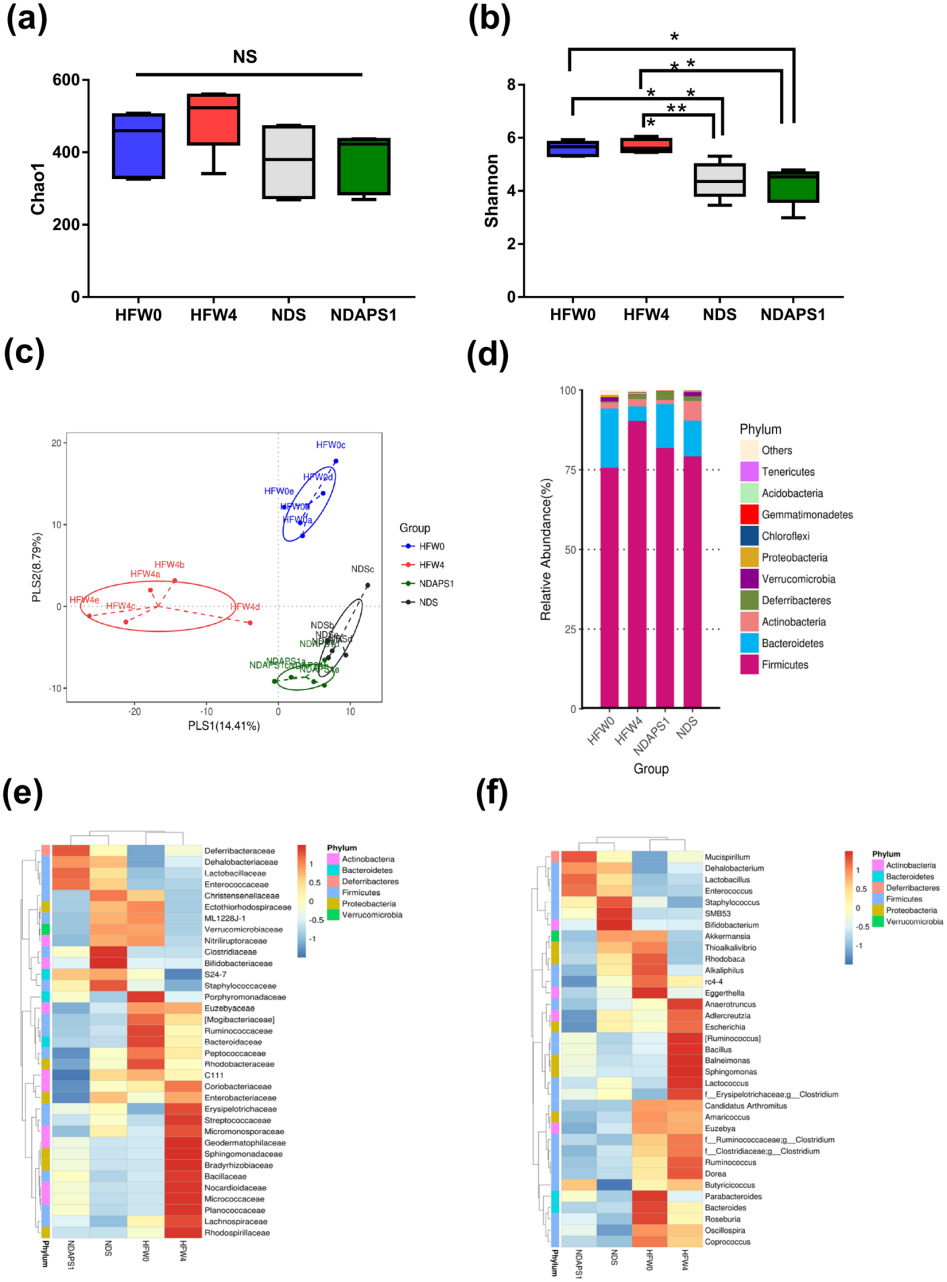
NDAPS1 manipulated gut microbiota in preexisting obese mice after dieting. (**a**) The chao1 richness estimator, (**b**) Shannon’s diversity index and (**c**) PLS-DA plots represented changes between samples collected from HFW0, HFW4, NDS and NDAPS1. NDAPS1 manipulated the abundance of the gut microbiota at the (**d**) phylum, (**e**) family and (**f**) genus levels. n = 5 per group. The nonparametric Wilcoxon signed rank test for paired data and Mann-Whitney U test for unpaired data (NDS vs. NDAPS1) were used. *P < 0.05, **P < 0.01, ***P < 0.001. NS: not significant.

We next investigated whether APS1 intervention affected the composition of the gut microbiota in the feces of the test mice, as the gut microbiota might be involved in the metabolic complications of obesity and associated with the acceleration of weight loss after dieting. The chao1 index (Fig. 4a) showed that the bacterial richness was no difference among the groups. Whereas, Shannon’s diversity index (Fig. 4b), indicating the bacterial α-diversity, showed a tendency to increase in the HFW4 group compared with the HFW0 group. Both NDS and NDAPS1 groups also have a significantly lower Shannon diversity than the HFW0 and HFW4 groups.

The partial least squares discriminant analysis (PLS-DA) was performed based on OTUs level to evaluate the variant of gut microbiota composition among the groups (Fig. 4c). PLS-DA plot showed that PLS 1 and PLS 2 explained 14.41% and 8.79% of variation of gut microbiota composition, respectively. A significant separation in PLS 1 and PLS2 between each group was observed (*P* < 0.05) (Supplemental Fig. S1). These findings indicated that the composition of gut microbiota, which has been assumed to be in a dysbiotic state during the obesity phase, could be altered to an intermediate configuration between dysbiotic and normal states.

**Figure 5:**
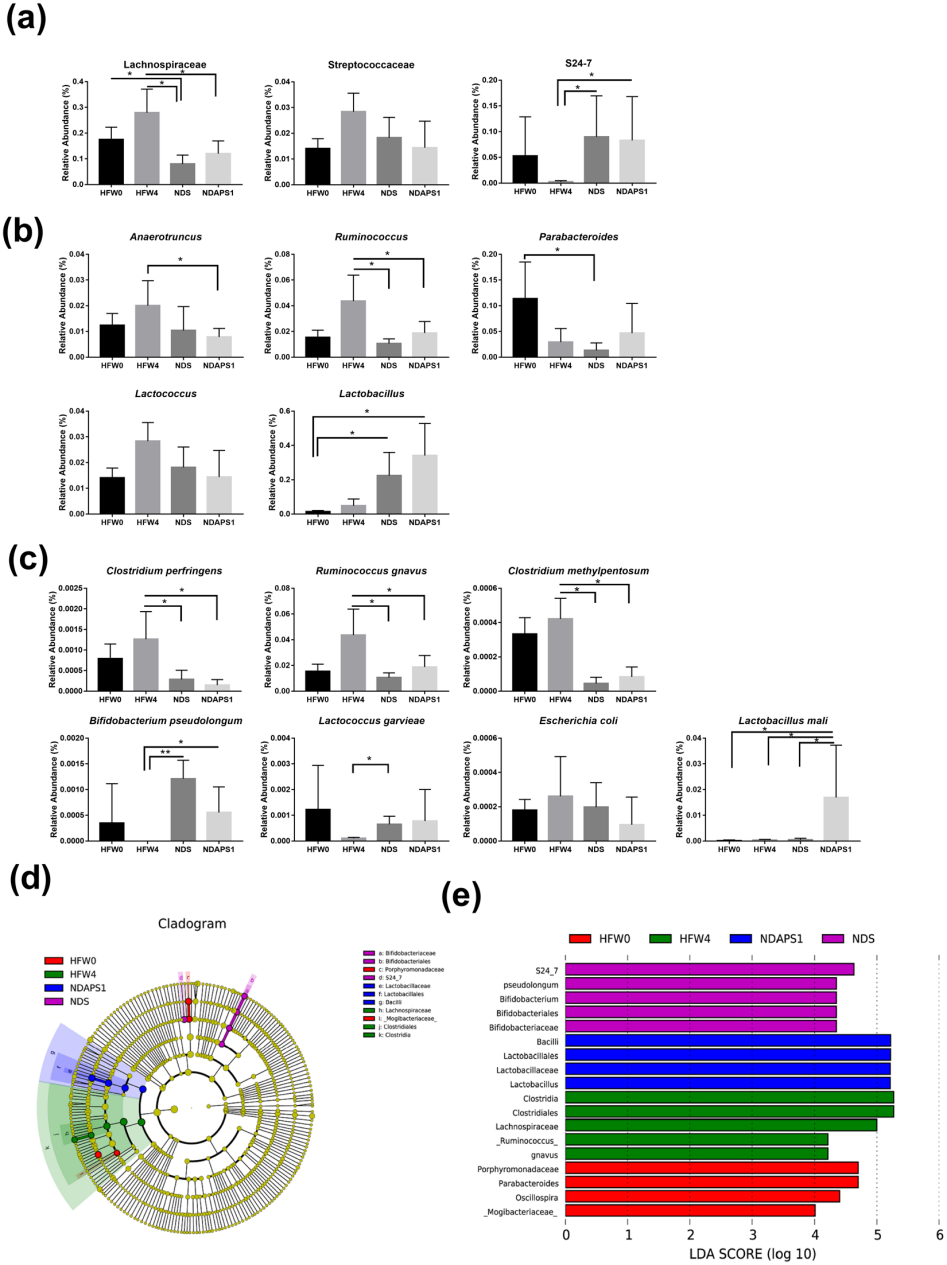
NDAPS1 manipulated gut microbiota in preexisting obese mice after dieting. NDAPS1 manipulated the abundance of specific bacteria at the (**a**) family, (**b**) genus and (**c**) species level. (**d**,**e**) LEfSe comparison of gut microbiota between HFW0, HFW4, NDS and NDAPS1 groups. n = 5 per group. The data are expressed as the mean ± SD. The nonparametric Wilcoxon signed rank test for paired data and Mann-Whitney U test for unpaired data (NDS vs. NDAPS1) were used. *P < 0.05, **P < 0.01, ***P < 0.001.

To further investigate the variability of gut microbiota between NDS and NDAPS1, a Venn diagram showing the overlaps between the groups was performed. The results indicated that 409 of the total richness of 751 OTUs were shared. Among shared OTUs, 74 OTUs and 127 OTUs were upregulated and downregulated, respectively, more than two folds in NDAPS1 when compared with NDS (Supplemental Fig. S2). Thus, we also analyzed the abundance of specific bacterial taxa to clarify the role of APS1 in alteration of intestinal microbiota. The results of the histogram of phyla (Fig. 4d) and heat maps of family (Fig. 4e) and genus (Fig. 4f) demonstrated very diverse patterns among treatments. After further taxonomic analysis, we observed that the mice in the diet period (NDS and NDAPS1 groups) had significantly reduced abundance of the Lachnospiraceae family, *Ruminococcus* genus and *Clostridium perfringens*, *Ruminococcus gnavus*, and *Clostridium methylpentosum* species (*P* < 0.05) and enhanced abundance of the S24_7 family and *Lactococcus garvieae*, and *Bifidobacterium pseudolongum* species when compared with obese mice (HFW4) (*P* < 0.05). Only APS1 intervention significantly increased the abundance of *Lactobacillus mali* and decreased the abundance of the Streptococcaceae family and *Anaerotruncus* genus (*P* < 0.05) (Fig. 5a–c).

Linear discriminant analysis effect size (LEfSe) was also calculated to further identify the specific bacterial taxa that were predominant in the 4 treatment groups. A total of 18 differentially abundant taxonomic clades (α = 0.05) were observed (Fig. 5d,e). The family Mogibacteriaceae (within the phylum Firmicutes) and genera *Parabacteroides* (within the phylum Bacteroidetes) and *Oscillospira* (within the phylum Firmicutes) were initially abundant in the HFW0 group. After HFD feeding for 4 weeks, the species *Ruminococcus gnavus* (within the phylum Firmicutes) became abundant. When on a diet, the most abundant taxa were the family S24_7 (within the phylum Bacteroidetes) and species *Bifidobacterium pseudolongum* (within the phylum Actinobacteria) in the NDS group. APS1 intervention specifically enriched the abundance of the *Lactobacillus* genus (within the phylum Firmicutes) in test mice.

We further used green fluorescent protein (GFP) to trace APS1 in the gastrointestinal tract of test mice (Supplemental Fig. S3a). After gavage for three hours, the results of immunofluorescence staining of cryostat sections confirmed that GFP-expressing APS1 was detectable in the terminal of the ileum of the mice, which is the region that *Lactobacillus* species frequently colonize^18^ (Supplemental Fig. S3b). These finding might indicate that accelerating weight loss by APS1 intervention in mice results in manipulating the intestinal microbiota with alteration of specific bacterial taxa.

### APS1 affected fecal SCFAs excretion and expression of satiety hormones in preexisting obese mice after dieting

**Figure 6:**
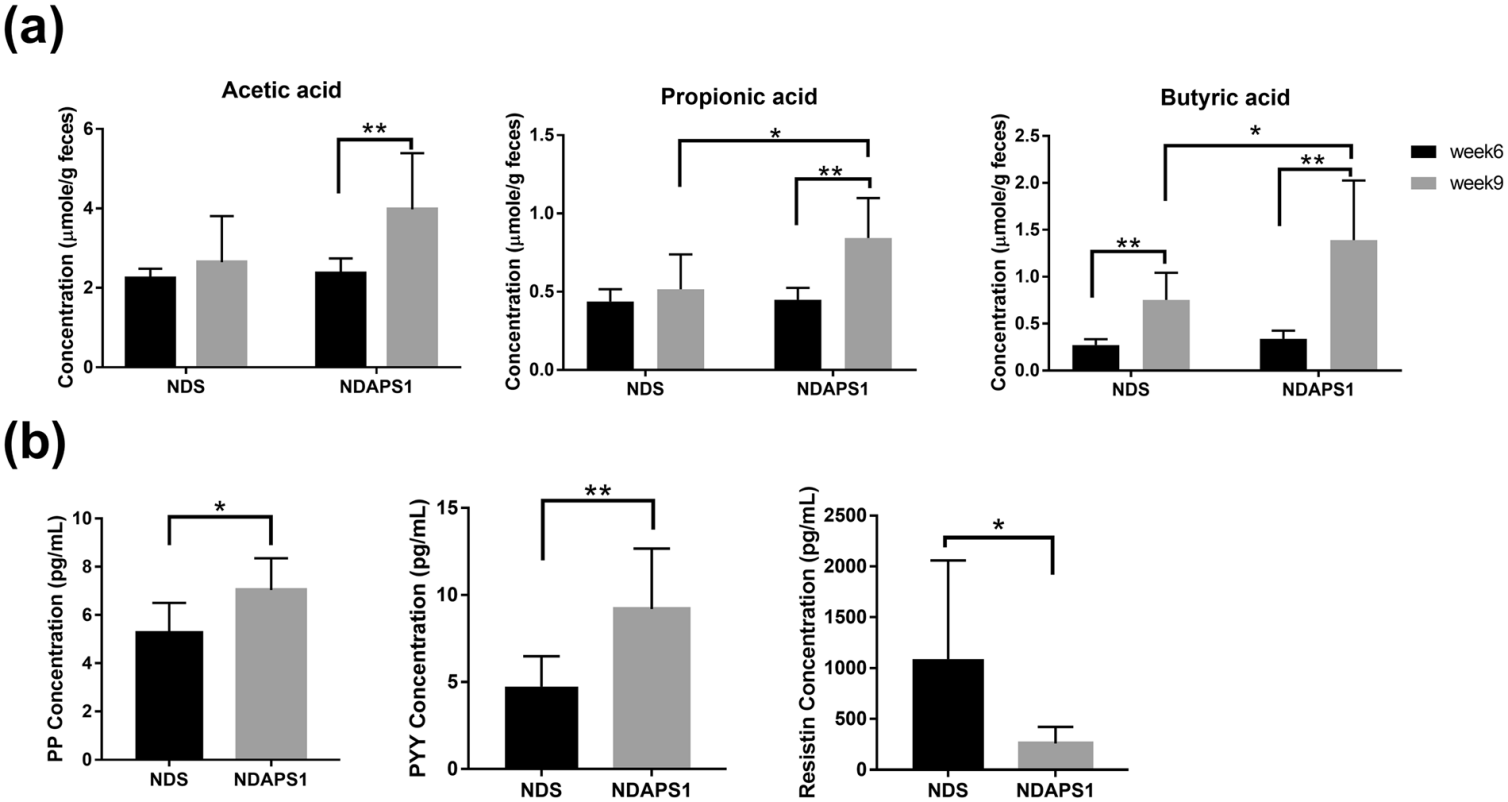
NDAPS1 increased fecal SCFA extraction and serum satiety hormone production in preexisting obese mice after dieting. (**a**) NDAPS1 increased SCFA extraction in feces of test mice at 6 weeks and 9 weeks. (**b**) NDAPS1 regulated satiety hormone production in the serum of test mice n = 8 per group. The data are expressed as the mean ± SD. Statistical analysis was conducted by using paired (week 6 vs. week 9) and unpaired (NDS vs. NDAPS1) two-tailed student’s t-test. *P < 0.05, **P < 0.01, ***P < 0.001.

Given the above causal connection between microbiota configuration and weight loss after dieting, we asked whether APS1 intervention could affect fecal SCFAs through alteration of gut microorganisms and further regulate the expression of satiety hormones to accelerate weight loss and ameliorate hepatic steatosis. The results indicated that the mice in the APS1 intervention significantly excreted higher fecal propionic acid and butyric acid levels compared to the NDS group at week 9 (*P* < 0.05) (Fig. 6a). We then investigated satiety hormones, PP, PYY and resistin, which are associated with appetite regulation. The results showed that in the mice that received the APS1 intervention, the production of serum PP (*P* < 0.05) and PYY (*P* < 0.01) were significantly induced and the production of resistin was significantly decreased (*P* < 0.05) compared with the NDS group after dieting (Fig. 6b).

### APS1-regulating metabolites contributed to accelerated weight loss in preexisting obese mice after dieting

**Figure 7:**
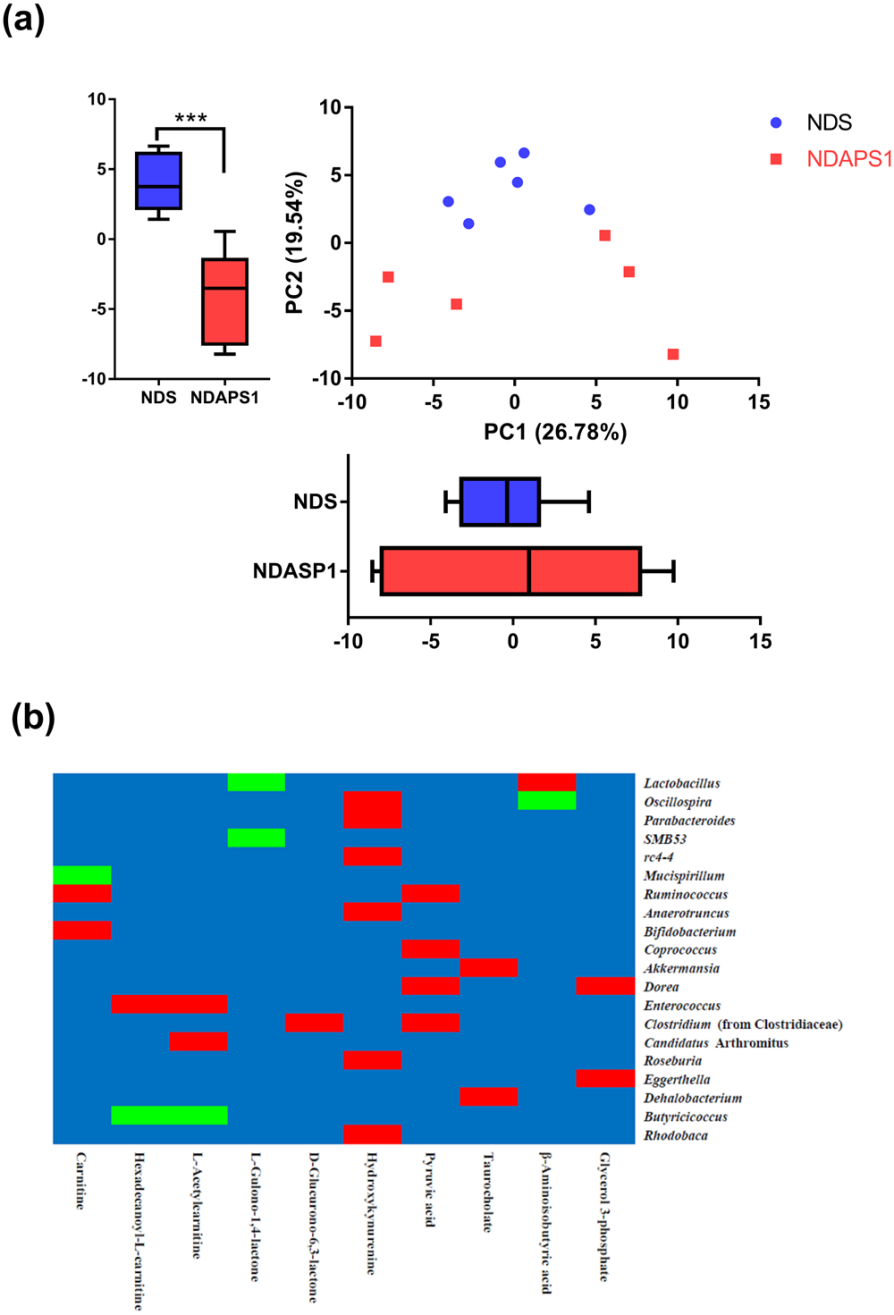
Two-dimensional PCA of metabolomics profile and Pearson’s correlation tests between predominant genera-level microbiota and metabolites. (**a**) PCA was based on PC1 and PC2 from mean intensity values of total detected metabolites in serum samples obtained from the NDS (solid blue circle) and NDAPS1 (solid orange square) groups. n = 6 per group. The data are expressed as the mean ± SD. Statistical analysis was conducted by using unpaired two-tailed student’s t-test. ***P < 0.001. (**b**) The relative abundance of bacterial taxa was significantly correlated with specific metabolites. Each cell was colored corresponding to Pearson’s correlation results. The significant positive correlation (*P* < 0.05) is represented as a red cell, and the significant negative correlation (*P* < 0.05) is represented as a green cell. The blue cells indicate the correlations were not significant (*P* > 0.05).

To further gain insight into how the APS1 intervention accelerated weight loss and ameliorated hepatic steatosis, we compared the serum metabolomics profiles of the NDS and NDAPS1 groups. The PCA plot showed that PC 1 and PC2 explained 26.78% and 19.54% of variation, respectively, with a significant difference in PC2 between NDS and NDAPS1 (*P* < 0.001) (Fig. 7a). Among the metabolites, 11 compounds showed significant differences between the NDS and NDAPS1 groups (*P* < 0.05) (Table 1). These metabolites were carnitine, hexadecanoyl-L-carnitine, L-gulono-1,4-lactone, L-acetylcarnitine, D-glucurono-6,3-lactone, hydroxykynurenine, pyruvic acid, taurocholate, D*-*glutamine, β-aminoisobutyric acid and glycerol 3-phosphate. After analyzing the possible metabolic pathways analysis using the KEGG database (Table 1), the APS1 intervention increased metabolites predominantly related to fatty acid degradation, ascorbate biosynthesis, conjugated bile acid biosynthesis and branched-chain amino acid degradation and reduced the triacylglycerol biosynthesis-associated metabolites in preexisting obese mice.

**Table 1:** Detected differential metabolites between NDS and NDAPS1 groups by LC-MS.

Metabolites	Ion(*m/z*)	Mean intensity		Fold change	*p*-value	Physiological function	Metabolic pathway
		NDS	NDAPS1	(NDAPS1/NDS)			
Carnitine	162.112	267596.07	441492.50	1.65	0.009	Fatty acid degradation	Lipid metabolism
Hexadecanoyl-L-carnitine	422.324	134.10	1892.88	14.12	0.017	Fatty acid degradation	Lipid metabolism
L-Gulono-1,4-lactone	177.040	199.88	1880.93	9.41	0.017	Ascorbate biosynthesis	Ascorbate and aldarate metabolism
L-Acetylcarnitine	204.123	845970.59	1018787.57	1.20	0.026	Insulin resistance	Lipid metabolism
D-Glucurono-6,3-lactone	175.024	12205.64	2117.76	0.17	0.026	Ascorbic acid synthesis	Ascorbate and aldarate metabolism
Hydroxykynurenine	225.086	1663.02	283.58	0.17	0.026	Amino acid biosynthesis	Tryptophan metabolism
Pyruvic acid	87.008	4556.93	332.37	0.07	0.026	Energy source	Citrate cycle
Taurocholate	514.284	1666.34	89307.51	53.60	0.041	Conjugated bile acid biosynthesis	Taurine and hypotaurine metabolism
D-Glutamine	169.058	6640.24	8602.06	1.30	0.041	Insulin and glucose modulation	D-Glutamine and D-glutamate metabolism
β-Aminoisobutyric acid	104.070	1459.53	3631.36	2.49	0.044	Branched-chain amino acid degradation	Amino acid metabolism
Glycerol 3-phosphate	171.006	1163.82	420.82	0.36	0.044	Triacylglycerol biosynthesis	Lipid metabolism

1 n = 6 per group.

2 Fold change was calculated by dividing the mean of the peak intensity of each metabolite from NDS and NDAPS1 groups.

3 Statistical analysis was conducted by using unpaired two-tailed student’s t-test.

Moreover, to explore the functional correlation between the NDAPS1-mediated gut microbiome and metabolites, the correlations among 20 predominant bacterial taxa at the genus level and 10 metabolites were calculated using Pearson’s correlation tests in Fig. 7b. *Ruminococcus*, *Bifidobacterium*, *Enterococcus* and *Candidatus* Arthromitus were positively correlated with fatty acid oxidation-related metabolites (including carnitine, hexadecanoyl-L-carnitine and L-acetylcarnitine), whereas *Mucispirillum* and *Butyricicoccus* were negatively correlated. Both *Lactobacillus* and *SMB53* were negatively correlated with L-gulono-1,4-lactone. *Clostridium* (from Clostridiaceae) was positively correlated with D-glucurono-6,3-lactone. *Oscillospira*, *Parabacteroides*, *rc4–4*, *Anaerotruncus*, *Roseburia* and *Rhodobaca* were positively correlated with hydroxykynurenine. *Ruminococcus*, *Coprococcus*, *Dorea* and *Clostridium* (from Clostridiaceae) were positively correlated with pyruvic acid. *Akkermansia* and *Dehalobacterium* were positively correlated with taurocholate. β-Aminoisobutyric acid was positively correlated with *Lactobacillus* and was negatively correlated with *Oscillospira*. Glycerol 3-phosphate was positively correlated with *Dorea* and *Eggerthella*.

## Discussion

In the present study, we demonstrated that administration of *Lb*. *mali* APS1, isolated from sugary kefir grain, could accelerate weight loss, improve the features of obesity and ameliorate hepatic steatosis in preexisting obese mice after dieting. Few similar papers have shown the effects of different probiotic interventions on weight loss during the dieting stage in rodent models and human pilot studies^19–23^. Among these studies, most results did not show significant effects, and only one study using *Lb*. *plantarum* showed a positive impact on weight loss^20^.

To further evaluate the possible mechanisms, we hypothesized that alteration of the microbiota configuration by APS1 intervention contributed to enhanced metabolic homeostasis through metabolite-mediated effects on host metabolism. The mechanism results highlighted three interdependent effects of the gut microbiome on acceleration of weight loss, metabolic modulation and hepatic lipid metabolism through APS1 intervention in combination with dieting.

First, the APS1 intervention induced specific changes in the diversity and abundance of obesity-associated bacteria in intestinal microbiota. A tendency to increase Shannon diversity index in HFW4 as compared with HFW0 was observed. Both NDS and NDAPS1 groups also showed a significantly lower Shannon diversity than HFW0 and HFW4. These findings differed from most studies on the obese individual showing the decrease in gut microbial diversity^24,25^. However, certain studies reported an increase in microbial diversity in HFD-fed rats^26^ and the diabetic subjects with high BMI^27^, which were parallel with our findings. The occasion of this inconsistency is unknown and remains to re-evaluate, but the differences in small sample size, diet, caloric intake or other conditions may influence the observation^28,29^. Moreover, the distribution of bacterial abundance is also a key factor affecting the bacterial diversity. The NDS and NDAPS1 groups demonstrated more than 10 and 25 folds in abundance ratios of *Lactobacillus* / *Clostridium* than HFW4, respectively, resulting in reduction of Shannon diversity index. This finding might suggest that both APS1 intervention and weight loss could promote the beneficial bacteria on competitive exclusion of specific deleterious bacteria.

The abundance of obesity-associated bacteria in intestinal microbiota was also affected by the APS1 intervention. The decreased abundances of Lachnospiraceae, *Ruminococcus* and *Clostridium* were found in both dieting groups (NDS and NDAPS1). The Lachnospiraceae family has been shown to have a positive correlation with inflammation markers in white adipose tissues and body weight gain in diet-induced obese mice^30^. *Ruminococcus* and *Clostridium* were butyrate producers, which were related to reduce dietary energy^31,32^. Uniquely, APS1 intervention regulated the levels of Streptococcaceae, *Anaerotruncus* and *Lactococcus*. Increasing the abundance of the Streptococcaceae family was associated with obesity, but it had also been shown that some strains in Streptococcaceae could also induce mild inflammation in humans, leading to the uneven morbidity related to chronic wounds among diabetic subjects^33^. *Anaerotruncus* was reported to be linked to hepatic cirrhosis with *Holdemania* and *Dorea* and type 1 diabetes, but not specific to insulin resistance, which was normally associated with HFD^34,35^. Interestingly, we found that the abundance of *Lactobacillus mali* was significantly increased in NDAPS1 group. This result reflected that the APS1 strain may survive in the intestine and associated with increased proportion of *Lactobacillus*.

Second, the APS1-manipulated gut microbiome influenced obesity-associated metabolites, regulated lipid metabolism, enhanced energy expenditure and inhibited appetite. Carnitine, hexadecanoyl-L-carnitine, L-acetylcarnitine, β-aminoisobutyric acid and glycerol 3-phosphate have been reported to be involved in the lipid/fatty acid metabolic pathway. Carnitine aids in a shuttle process that makes long-chain fatty-acid coenzyme A derivatives available for β-oxidation, leading to increasing ketogenesis and lipolysis^36^, whereas other long-chain acyl-carnitines could be metabolic intermediates of the fatty acid incorporation pathway into erythrocyte membrane phosphatidylcholine, and phosphatidylethanolamine^37^. The secretion of β-aminoisobutyric acid can be triggered by increasing PGC1α protein expression in skeleton myocytes after exercise, resulting in activation of β-oxidation in hepatocytes and induction of brown adipocyte-specific gene expression in white adipocytes *in vitro* and *in vivo*^38^. Additionally, plasma β-aminoisobutyric acid could be increased by the degradation of branched-chain amino acids, which are associated with insulin resistance in obesity^39^. L-Glycerol 3-phosphate is a recognized precursor of glyceride glycerol. A rise in the concentration of L-glycerol 3-phosphate contributes to an increase in fatty acid esterification and triacylglycerol biosynthesis^40^. Glutamine, one of the most effective substrates for gluconeogenesis, is the major gluconeogenic precursor in the kidney^41^. The glucose-lowering effect of glutamine could be linked to the known effect of glutamine on fat metabolism such as inhibition of fatty acid oxidation and lipolysis^42^. A previous study reported that the reduction in serum glutamine concentration in obese individuals was associated with an increase in certain bacteria, such as *Ruminococcus* spp. and *Dorea longicatena*, which produce glutamate from glutamine^43^. An increase in serum taurocholate was associated with weight loss and energy expenditure by cholic acid treatment *in vivo*^44^. Taurocholate stimulating secretion of glucagon-like peptide-1 and PYY modulated insulin resistance and glucose metabolism in obese patients^45,46^.

We further revealed that several bacterial taxa were positively or negatively correlated with APS1-mediated specific metabolites in test mice. For instance, *Akkermansia* was found to be positively correlated with taurocholate, which is synthesized via oxidation of cholesterol in the liver. This finding was similar to one indicating that *Akkermansia muciniphila* was negatively associated with serum cholesterol and low-density lipoprotein cholesterol levels in postmenopausal women with obesity^47^. Dysbiosis of *Oscillospira* leading to obesity and metabolic disorder *in vivo*^48^ might positively and negatively correlate with hydroxykynurenine and β-aminoisobutyric acid, respectively. Based on our results, we propose that the association between specific gut microbiota and metabolites might be used for the prediction of metabolic disease.

In addition to serum metabolites, intestinal metabolites, propionic acid and butyric acid, were also modulated by APS1 supplement and then contributed to increased energy expenditure and suppressed appetite. The intestinal SCFAs, especially butyrate, could increase energy expenditure through increasing mitochondrial respiration in mice^49^. SCFAs also play crucial roles in the gut-brain axis in the host^50^. They could stimulate the secretion of satiety hormones PYY and PP, which can regulate anorexigenic signaling in appetite^51^. Besides, higher SCFA excretion and increased PYY and PP expression in the NDAPS1 group than the NDS group might be due to an increase in the populations of *Lactobacillus* spp.^52,53^.

Third, APS1-manipulated gut microbiome influenced hepatic-associated metabolites, followed by the amelioration of hepatic steatosis through the gut-liver axis. D-glucurono-6,3-lactone, glycerol-3-phosphate, and pyruvic acid, which are associated with fatty liver disease^54^, have been identified as intermediates of TG synthesis in the liver tissues of mice on an HFD^55^. It is plausible that the ameliorating hepatic steatosis effect of APS1 might be partially due to the lower serum levels of certain fatty liver-associated metabolites. Additionally, butyrate plays important roles in the gut-liver axis in the host^50^. Butyrate could be transferred from the intestine to the liver via the portal vein and activate SIRT1 and induce SIRT1-dependent PGC1 deacetylation through AMPK activation^56^. SIRT1 has been shown to modulate lipid metabolism via activating PGC1 and upregulation of fatty acid oxidation genes^57^.The activation of SIRT1/PGC1 could further suppress the expression of lipid synthesis factors, FAS and ACC, and fatty acid transporter FABP4, and thus limited the accumulation of fatty acids^58^. The results of protein expression including upregulation of SIRT1 and PGC1 and downregulation of FAS and FABP4 in liver tissue of the NDS group provided evidences to support amelioration of hepatic steatosis of APS1 might through the gut-liver axis. Therefore, suppressing in hepatic steatosis may attribute to APS1-induced butyrate expression triggering hepatic protective functions of SIRT1.

Finally, our study provides an example of how probiotics could serve as a potential therapy for modulating physiological function downstream of the microbiota. We found that APS1-induced modulation of SCFAs and circulating metabolites enhanced susceptibility to accelerated weight loss and ameliorated hepatic steatosis, potentially through improvement of energy expenditure and inhibition of appetite. Future studies are warranted that examine the potential clinical use of APS1 as a novel therapeutic in the quest for effective long-term weight management solutions.

## Material and Methods

### Bacterial strain

*Lactobacillus mali* APS1 was isolated and identified from sugary kefir^59^. The preparation of APS1 followed the procedure previously described^17^.

### Animal experimental protocol

Male C57BL/6 J mice at 7 weeks of age were used (*BioLASCO Taiwan* Co., Ltd, Yi-Lan, Taiwan). Test mice were housed in cages (one mouse per cage) at a controlled room temperature and under a 12 h light–dark cycle and provided food and filtered water *ad libitum*. After one week for acclimatization, all test mice were fed 60% calorie fat HFD (D12492, Research Diets, Inc., NJ, USA) for 6 weeks, at which point mice showed obesity phenotype characteristics^60^. After HFD feeding, test mice were then treated for the following three weeks and were randomized into two groups: control group (NDS) received 10% calorie fat normal diet (D12450B, Research Diets, Inc.) administered with saline by gavage daily, and the APS1 intervention group (NDAPS1) received a normal diet and APS1 at 5 × 10^8^ CFU in saline by gavage daily. The body weight and food intake of the test mice were recorded once a week. The cumulative caloric intake was calculated by the total gram weight of diet taken multiplied by the caloric content of the diet (ND: 16.11 kJ/g; HFD2:1.92 kJ/g). At the end of the experiment, all test mice were fasted for 8 hours, anesthetized with isoflurane and sacrificed for serum and tissue collection. All experiments were performed in accordance with relevant guidelines, the animal experimental protocol was approved by the Institutional Animal Care and Use Committee of the National Taiwan University (Approval No: NTU103-EL-00096).

### Analysis of serum biochemistry and satiety hormones

The serum samples were collected from test mice and stored at −80 °C until analysis. Serum glucose, triglyceride (TG) and total cholesterol (T-Chol) levels were measured using a Fuji DRI-CHEM Clinical Chemistry Analyzer FDC 3500 (Fujifilm, Tokyo, Japan) at the National Laboratory Animal Center (Taipei, Taiwan). Serum insulin level was measured using a mouse insulin assay kit following the manufacturer’s protocol (Mercodia, Uppsala, Sweden). Homeostasis model assessment of insulin resistance (HOMA-IR) index was calculated according to the formula: HOMA-IR = insulin (μU/mL) × glucose (mg/dL)/405. Serum peptide YY (PYY), pancreatic polypeptide (PP) and resistin levels were measured using the Milliplex® MAP Kit and Mouse Metabolic Hormone 96-Well Plate Assay (Merck Millipore, MA, USA).

### Analysis of fecal SCFAs

Fecal samples were collected from test mice at 6 weeks and 9 weeks. SCFAs in feces were extracted with 70% ethanol solution and analyzed using published methods^61^. Briefly, the samples were processed to fatty acid derivatization and then extracted twice with ether. Each ether layer was collected and concentrated under nitrogen gas. The obtained fatty acid hydrazides were analyzed by HPLC.

### Histological analysis

Liver and white adipose tissues (epididymal, retroperitoneal and inguinal white adipose tissue; namely, eWAT, rpWAT and iWAT) were obtained from test mice and fixed with 4% formalin overnight and then embedded within paraffin or optimal cutting temperature (OCT) compound for frozen section procedures. Tissue sections (5-μm thickness) were stained with hematoxylin and eosin (H&E) or Oil Red O (O0625, Sigma-Aldrich, MO, USA) staining. Histological images were obtained under a microscope (AxioObserver Z1, Göttingen, Germany). The mean adipocyte size was measured using ImageJ software (NIH, MD, USA).

### Western blot analysis

Total protein from the liver tissues of mice was extracted with RIPA buffer containing protease and phosphatase inhibitors. The procedures for electrophoresis and blotting were conducted following described methods^62^ with some modifications. The primary antibodies, including anti-SIRT1 (D1D7, Cell Signaling, MA, USA), anti-acetyl-CoA carboxylase (ACC) (C83B10, Cell Signaling), anti- peroxisome proliferator-activated receptor gamma coactivator 1 (PGC1) (ab54481, Abcam, Cambridge, UK), anti-fatty acid synthase (FAS) (C20G5, Cell Signaling), anti-fatty acid binding protein 4 (FABP4) (D25B3, Cell Signaling) and anti-β-actin (13E5, Cell Signaling) antibodies, were used for identification of each protein. Quantification of protein levels in luminescent bands was performed using ImageJ software.

### Analysis of hepatic triglyceride

Hepatic triglyceride levels were measured using a triglyceride colorimetric assay kit (Cayman Chemical, MI, USA). Briefly, weighed liver tissues were homogenized in cold buffer on ice. The homogenates were centrifuged at 10,000 g for 15 minutes at 4 °C. Supernatants were harvested and stored at −80 °C for further assay following the manufacturer’s protocol.

### DNA extraction and sequencing of gut microbiota

Fecal samples were collected from test mice at 0 weeks (HFW0) and 4 weeks (HFW4) during HFD feeding, and at 3 weeks after switching to an ND (NDS and NDAPS1, respectively). Bacteria total genomic DNA from fecal samples was extracted using published methods^63^ with modifications. The next-generation sequencing results of bacterial 16 S ribosomal DNA genes were then analyzed following procedures described previously^64^. Although, different variable regions of 16 s rRNA have been targeted for distinguishing the bacteria^65,66^, the V3-V4 region has been identified as the most useful for distinguishing among species of the intestinal bacteria^67,68^. This region is generally used for intestinal microbiome studies with accumulation of information and database. Therefore, the V3–V4 regions of 16S rRNA genes were amplified using a specific primer with a barcode. Barcoded PCR amplicons were sequenced on an Illumina HiSeq. 2500 platform. Operational taxonomic unit (OTU) clustering and species annotation were performed from representative sequences using UPARSE software (Version 7.0.1001) and the Greengenes Database based on Ribosomal Database Project classifier (Version 2.2), respectively. For reads assembly and quality control, paired-end reads was assigned and based on their unique barcode and truncated by cutting off the barcode and primer sequence. Paired-end reads were merged using FLASH (V1.2.7)^69^. Quality filtering on the raw tags was performed under specific filtering conditions according to the Qiime (V1.7.0)^70,71^. The tags were compared with the reference database (Gold database) using UCHIME algorithm^72^ to detect and remove chimera sequences^73^. For OTU cluster and species annotation, sequences analysis were performed by Uparse software (Uparse v7.0.1001)^74^. OTUs were assigned at sequences ≥97% similarity threshold. The GreenGene Database^75^ was used based on RDP classifier (Version 2.2)^76^ algorithm to annotate taxonomic information. OTUs abundance information was normalized using a standard of sequence number corresponding to the sample with the least sequences. Prior to various analyses, each sample was rarefied to an even sequence depth of 31,315 to control the various sequencing depth. Subsequent analysis of alpha diversity and beta diversity were all performed according to this output normalized data. The alpha diversity (Chao 1 and Shannon index) and PLS-DA were calculated with QIIME (Version 1.7.0) using R software (Version 2.15.3).

### Analysis of serum metabolomic profile

The metabolomic profile experiments were conducted by the commission service of the Metabolomics Core Laboratory at Center of Genomic Medicine, National Taiwan University (Taipei, Taiwan). Briefly, serum samples were collected from test mice and stored at −80 °C until analysis. Then, 100 μL of each serum sample was extracted for 2 minutes in 400 μL of ice-cold methanol, followed by vaporization. The vaporized serum residues were reconstituted with 200 μL of 50% methanol and then subjected to UHPLC-QTOF-MS analysis. The untargeted metabolic profile was analyzed using an Agilent 1290 UHPLC system (Agilent Technologies, CA, USA) coupled with an Agilent 6540 quadrupole-time of flight mass spectrometer (Agilent Technologies, CA, US) with electrospray ionization. The Acquity HSS T3 column (2.1 × 100 mm, 1.8 μm, MA, USA) was used and maintained at 40 °C. For analysis of metabolomics mass spectra, the mass spectra data were analyzed using TIPick^77^, XCMS2^78^ and Batch Normalizer^79^ methods.

### Analysis of the correlation between the gut microbiome and specific metabolites

The correlation relationships between the relative abundance of the gut microbiota at the genus level and 10 specific metabolites were evaluated by Pearson’s correlation test using SAS version 9.4 software (SAS Institute Inc., Cary, NC, USA) following a previously described method with modifications^80^. All metabolite data were mean-centered and scaled to unit variance prior to the test.

### Statistical analysis

All values are given as the mean ± standard deviation (SD). The data were proved to be normally distribution by using a Shapiro-Wilk test for normality (*P* > 0.05). Statistical analysis for animal experiments comparing two groups used paired and unpaired, two-tailed Student’s t-test. *P* < 0.05 was indicative of a significant difference. To perform and compare the statistical significant difference between two groups without a normal distribution assumption in NGS analysis, NGS data were normalized and the nonparametric Wilcoxon signed rank test for paired data and Mann-Whitney U test for unpaired data were used. LEfSe analysis with LDA scores greater than 4 and significance at α < 0.05 was determined by Kruskal–Wallis test. For All statistical analyses were performed using Statistical Analysis System software 9.4.

## Electronic supplementary material

Supplementary information
