# Supplementary material for: A combination of Lactobacillus mali APS1 and dieting improved the efficacy of obesity treatment via manipulating gut microbiome in mice
Source: Sci Rep. 2018 Apr 18;8:6153. doi: 10.1038/s41598-018-23844-y (PMC5906640; doi:10.1038/s41598-018-23844-y)
Supplement: Supplementary file 1 — Supplementary information [file 41598_2018_23844_MOESM1_ESM.docx]

**A combination of *Lactobacillus mali* APS1 and dieting improved the efficacy of obesity treatment via manipulating gut microbiome in mice**

**Yung-Tsung Chen^1#^, Ning-Sun Yang^1,2#^, Yu-Chun Lin ^3,4^, Shang-Tse Ho^3^, Kuan-Yi Li^3^, Jin-Seng Lin^5^, Je-Ruei Liu^1,3^, Ming-Ju Chen^3,^***

^1^ Institute of Biotechnology, National Taiwan University, Taipei City, 106, Taiwan

^2^ Agricultural Biotechnology Research Center, Academia Sinica, Taipei City 115, Taiwan

^3^ Department of Animal Science and Technology, National Taiwan University, Taipei City, 106,

^4^ Taiwan Livestock Research Institute, Council of Agriculture, Executive Yuan, Tainan City, 71246, Taiwan

^5^ SynbioTech Incorporation, Kaohsiung City, 821, Taiwan

*Corresponding authors:

Ming-Ju Chen at:

Department of Animal Science and Technology, National Taiwan University, No.50, Ln. 155, Sec. 3, Keelung Rd., Da’an Dist., Taipei City 106, Taiwan

TEL: 886-2-33664169

FAX: 886-2-2732-4070

E-mail: [cmj@ntu.edu.tw](mailto:cmj@ntu.edu.tw)

# These authors contributed equally to this work.

**Supplementary information**

**Supplementary material and methods**

**Generation of GFP-expressing APS1**

The pSLP256gfp plasmid DNA carrying green fluorescent protein gene was kindly provided from Dr. Bor-Rung Ou at TungHai University in Taiwan. For plasmid propagation, *Escherichia coli* DH5α was used and grown in Luria Bertani (LB) medium (Acumedia, NEOGEN Co., MI, USA) at 37℃. The pSLP256gfp plasmid DNA was purified using the EasyPure Plasmid Midi Advanced Kit (Bioman Scientific Co., Taipei, Taiwan) following the manufacturer’s protocol. The transformation of APS1 by electroporation was carried out following the method described previously[^1^](#_ENREF_1). Briefly, APS1 were cultured in fresh MRS medium (Acumedia) containing 2% glycine until reaching the optimal concentration. The cultured cells were harvested and washed twice with ice-cold buffer (952 mM sucrose and 3.5 mM MgCl_2_．6H_2_O). Before electroporation, 15 μg plasmid DNA was mixed with cells in buffer on ice. Electroporation was performed by using a MicroPulser electroporator (Bio-Rad, Hemel Hempstead, UK). The electroporated cells were than grown on MRS agar plates containing antibiotic 5 μg/mL of erythromycin for selection.

**Intestinal location of GFP-APS1 by immunofluorescence analysis**

Male C57BL/6J mice at 8 weeks of age were used and administered GFP-APS1 at 10^9^ CFU by gavage. Before and after administration for 3 hours, test mice were anesthetized with isoflurane and sacrificed for ileum tissue collection. The ileum tissues were fixed with 4% formalin overnight and then embedded within OCT compound for frozen sectioning. For immunofluorescence analysis, rabbit anti-GFP antibodies (Cell Signaling) were used as the primary antibody, and Cy5-conjugated anti-rabbit IgG antibody (Abcam) was used as secondary antibody. The fluorescent images were photographed using an LSM 510 META Laser Scanning Microscope (Zeiss, Heidelberg, Germany).

**Supplementary figures**

**
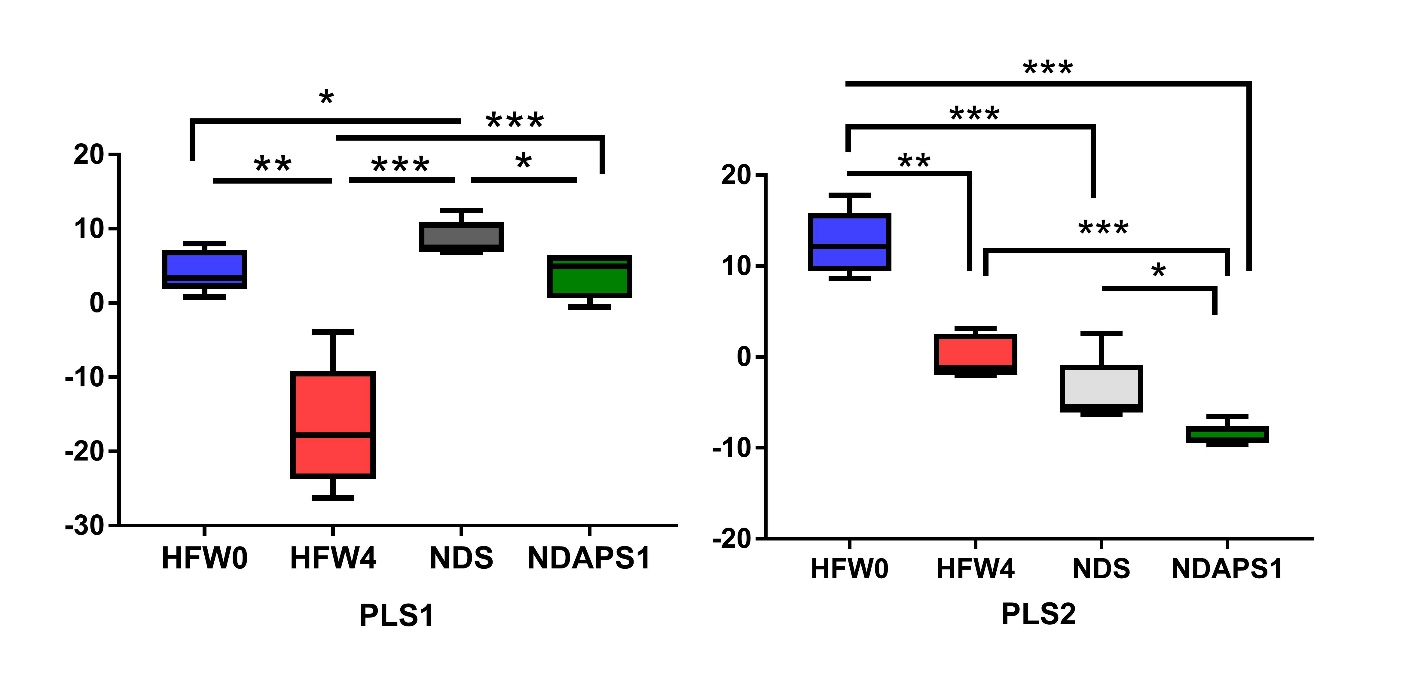
**

**Supplementary Figure S1**. Responses of the gut microbiota composition to HFW0, HFW4, NDS and NDAPS1 groups. The variation in PLS1 and PLS2 of PLS-DA plot among groups was performed. Statistical analysis was conducted by using paired and unpaired (NDS vs. NDAPS1) two-tailed student’s t-test. * *P* < 0.05, ** *P* < 0.01, *** *P* < 0.001.

**
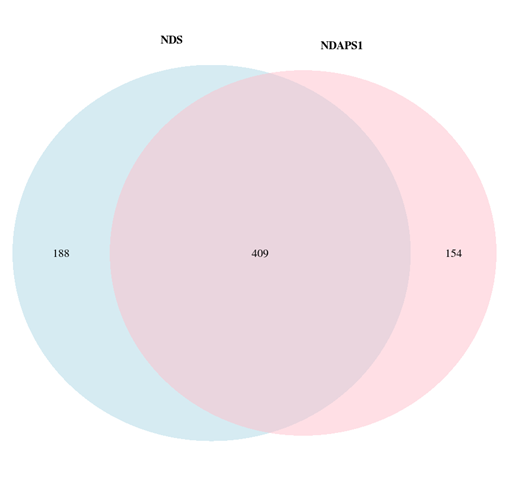
**

**Supplementary Figure S2**. Venn diagram showing the overlap of OTUs in gut microbiota among the NDS and NDASP1 groups.


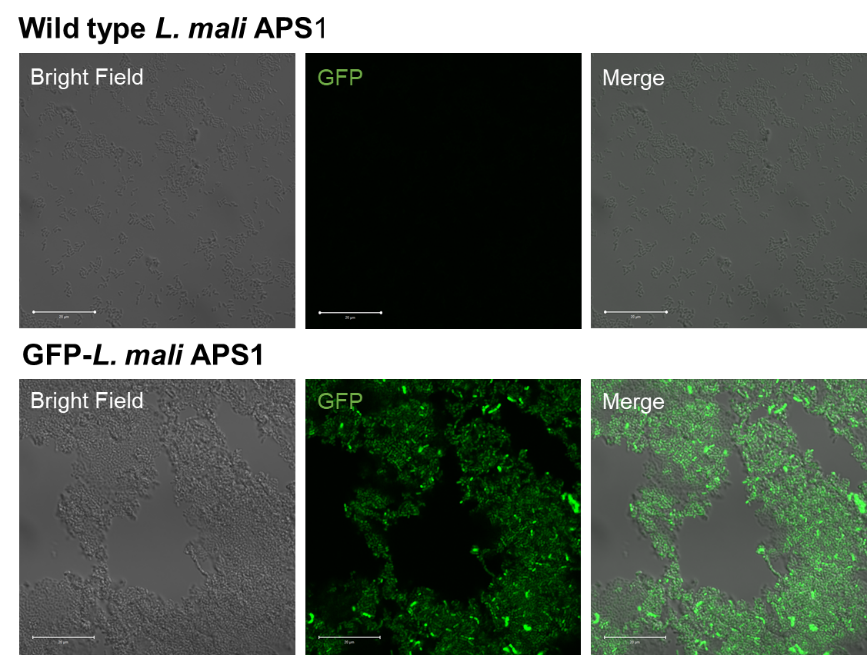


(b)


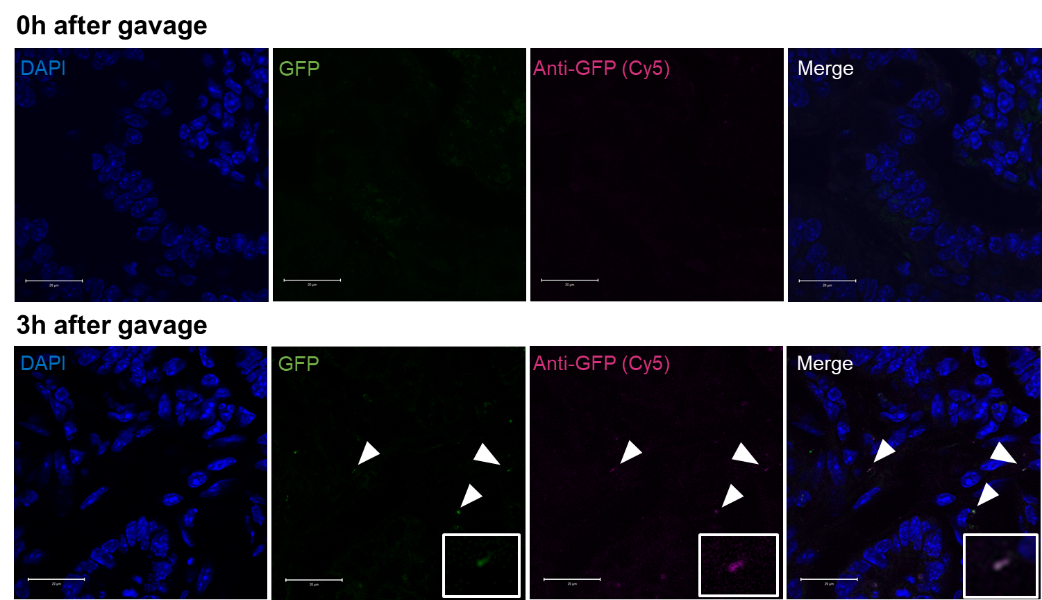


**Supplementary Figure S3**. Using GFP expression to trace the colonization of APS1 in the terminal of ileum of mice. (a) Representative wild field (left panel) and fluorescent (middle panel) images of GFP-APS1. (b) Immunofluorescent detection of GFP-positive bacteria (anti-GFP antibody in red) and GFP-APS1 (green) on DAPI-counterstained (nuclei in blue) cryosections of ileum from mice after gavage with 10^9^ CFU of GFP-APS1 for 0h and 3h, respectively. Arrows point co-localizations. The fluorescence of GFP-APS1 was detected by confocal laser scanning microscope. Scale bar indicates 25 µm.

Figure 3d, SIRT1 Figure 3d, PGC1


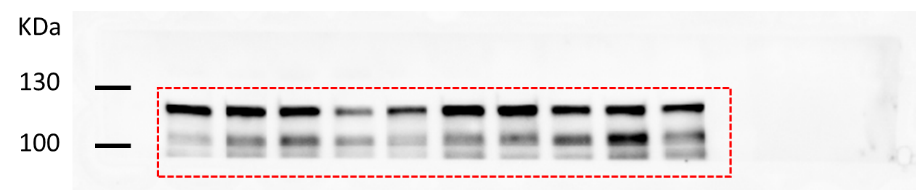

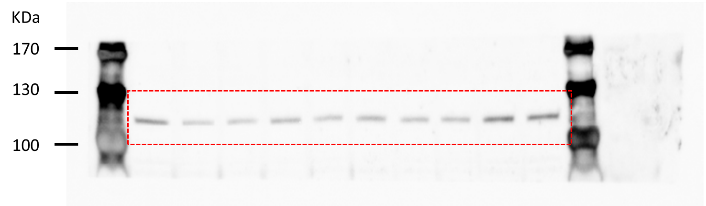


Figure 3d, ACC Figure 3d, β-actin


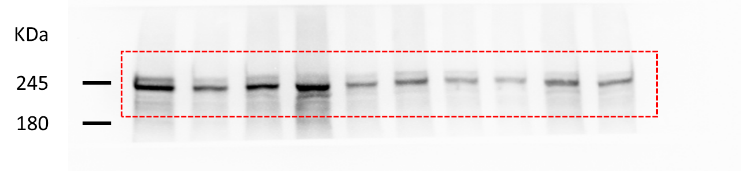

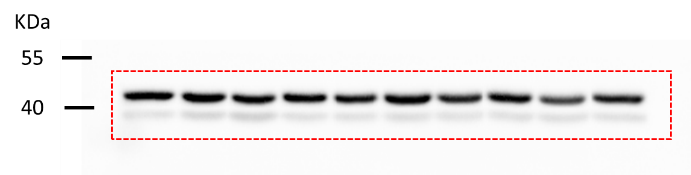


Figure 3d, FAS Figure 3d, FABP4


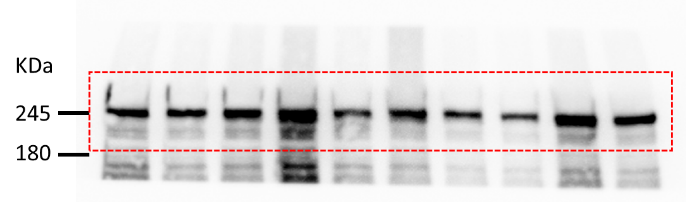

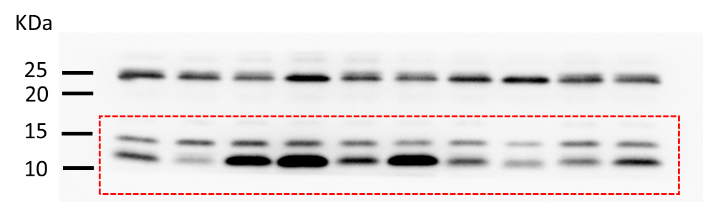


Figure 3d, β-actin


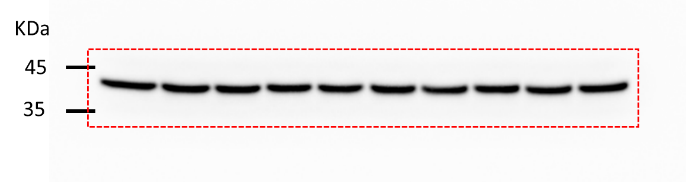


**Supplementary Figure S4**. Un-cropped full length images of blots in the [Figure](https://www.nature.com/articles/nsmb.2829/figures/6) 3d. Red dotted lines indicated the cropping area.

**Supplementary table**

**Supplementary TableS1.** Diet composition

|  | Control diet (D12450B) | High fat diet  (D12492) |
| --- | --- | --- |
| Energy (kJ%) | | |
| Protein | 20 | 20 |
| Carbohydrate | 70 | 20 |
| Fat | 10 | 60 |
| Total | 100 | 100 |
| kJ/gm | 16.11 | 21.92 |
| Ingredient (kJ) | | |
| Casein 30 Mesh | 3347.20 | 3347.20 |
| l-Cystine | 50.21 | 50.21 |
| Corn starch | 5271.84 | 0.00 |
| Maltodextrin 10 | 585.76 | 2092.00 |
| Sucrose | 5857.60 | 1151.44 |
| Soybean oil | 941.40 | 941.40 |
| Lard | 753.12 | 9225.72 |
| Vitamin Mix V10001 | 167.36 | 167.36 |
| Total | 16974.49 | 16975.32 |

**Supplementary reference**

1 Aukrust, T. W., Brurberg, M. B. & Nes, I. F. in *Electroporation Protocols for Microorganisms* (ed Jac A. Nickoloff) 201-208 (Humana Press, 1995).
